# Supplementary material for: PAI-1 mediates acquired resistance to MET-targeted therapy in non-small cell lung cancer
Source: PLoS One. 2024 May 17;19(5):e0300644. doi: 10.1371/journal.pone.0300644 (PMC11101109; doi:10.1371/journal.pone.0300644)
Supplement: S1 Fig — (PDF) [file pone.0300644.s001.pdf]

Figure 1D

EBC-1

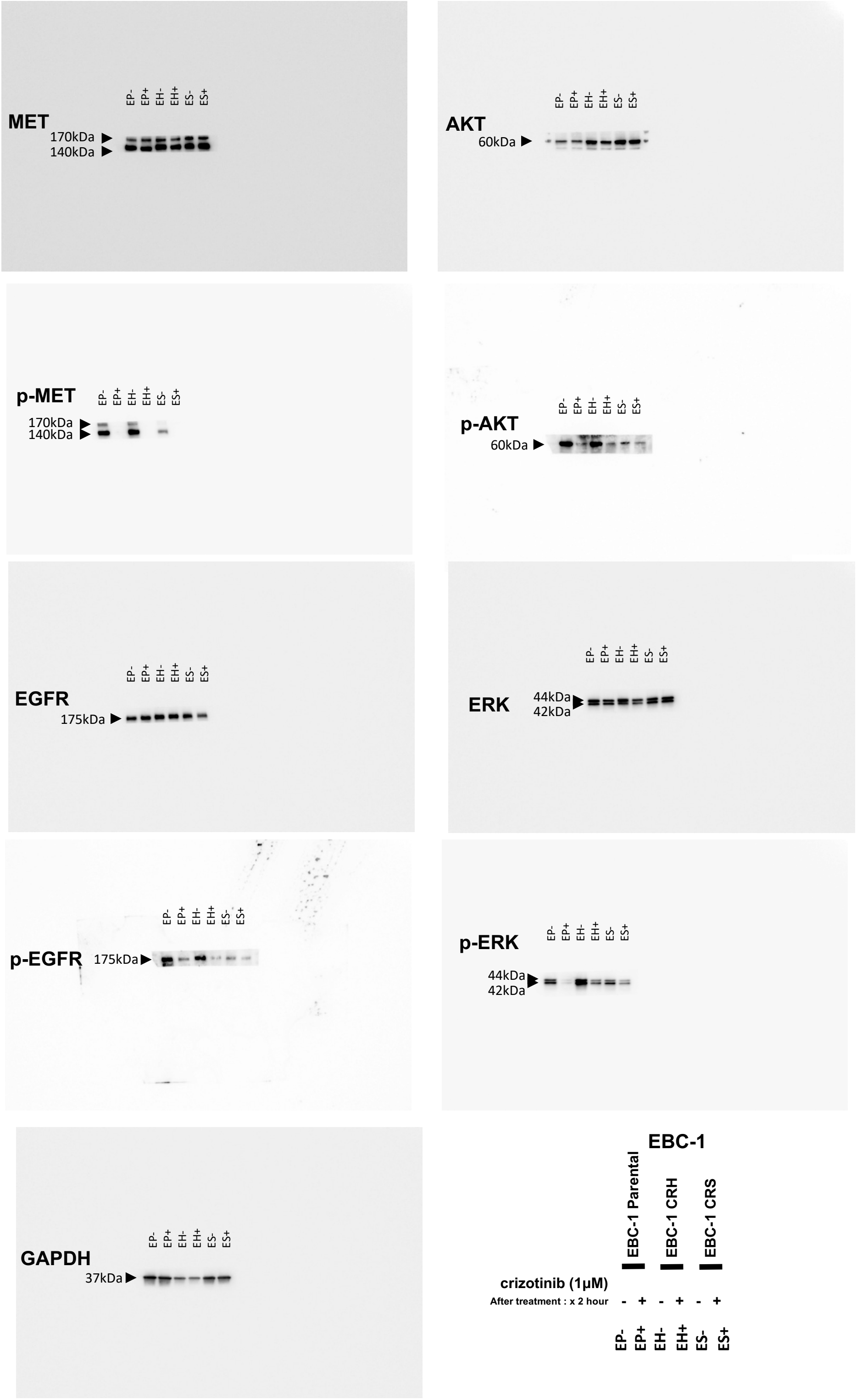

Figure 1D

H1993

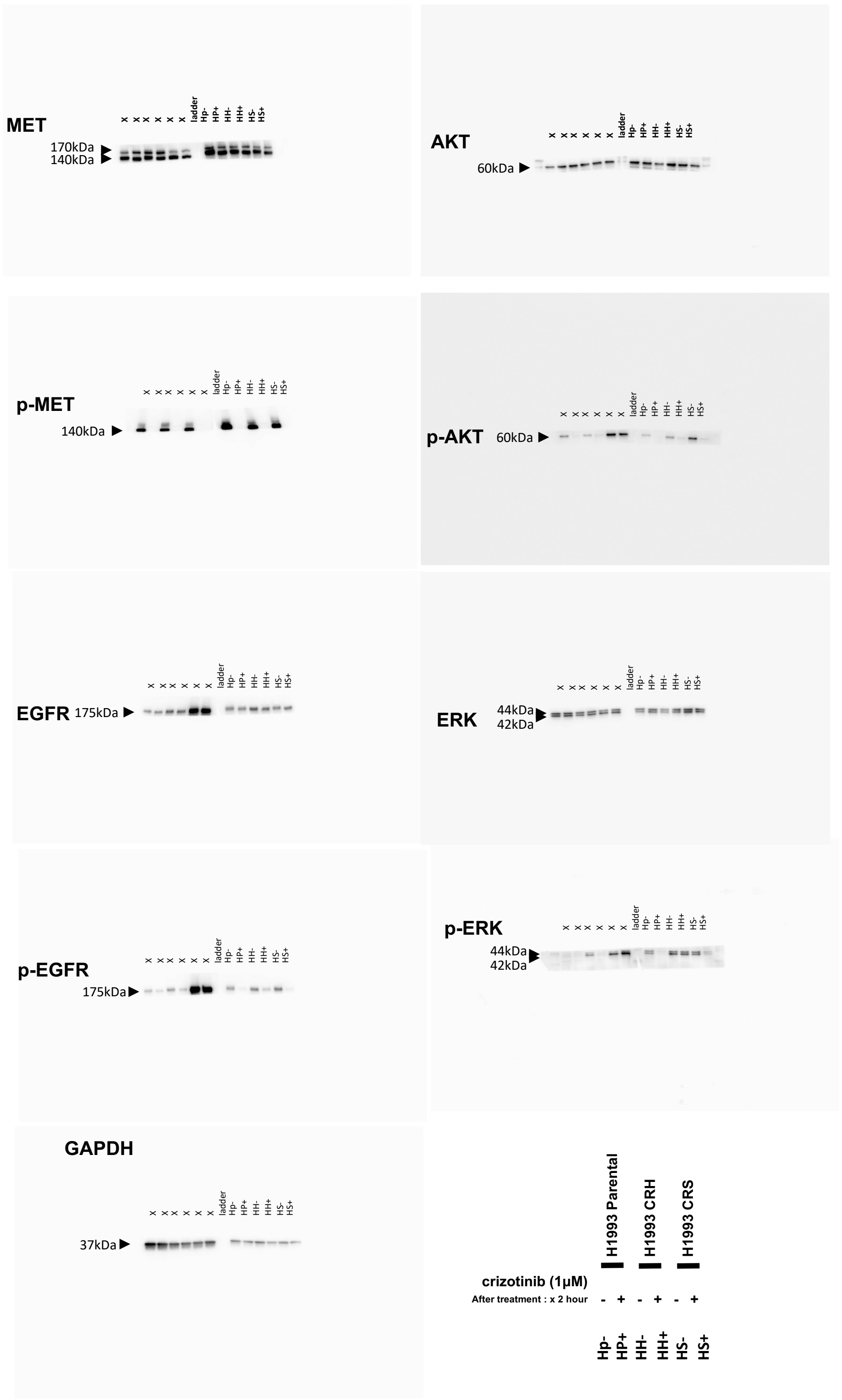

Figure 2B

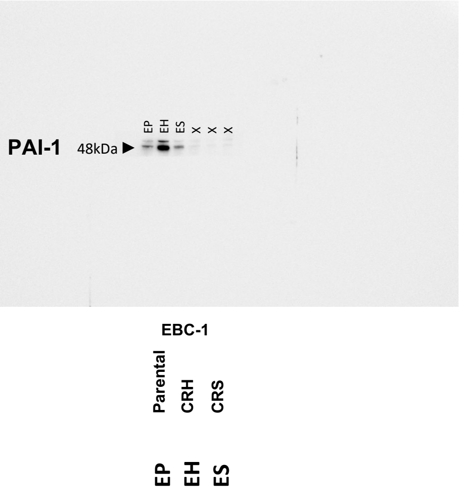

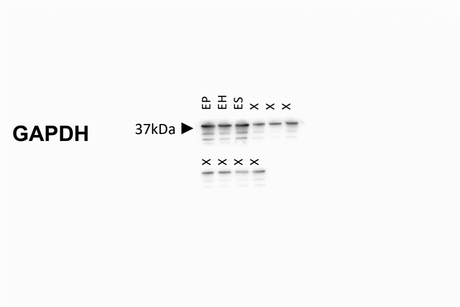

Figure 2E

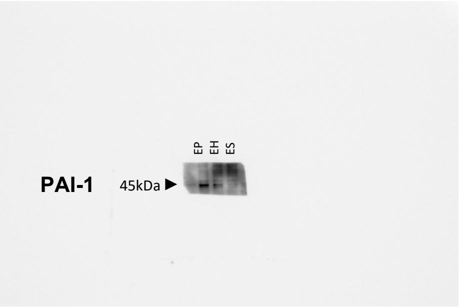

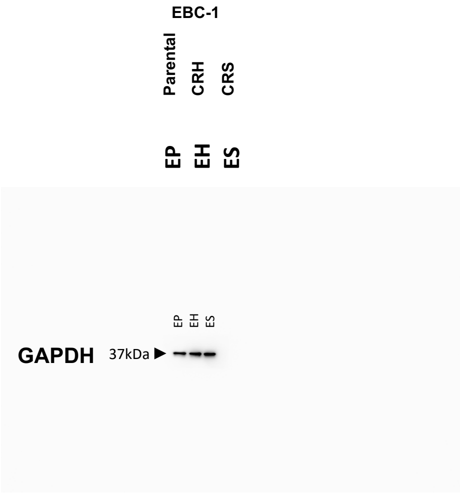

Figure 3A

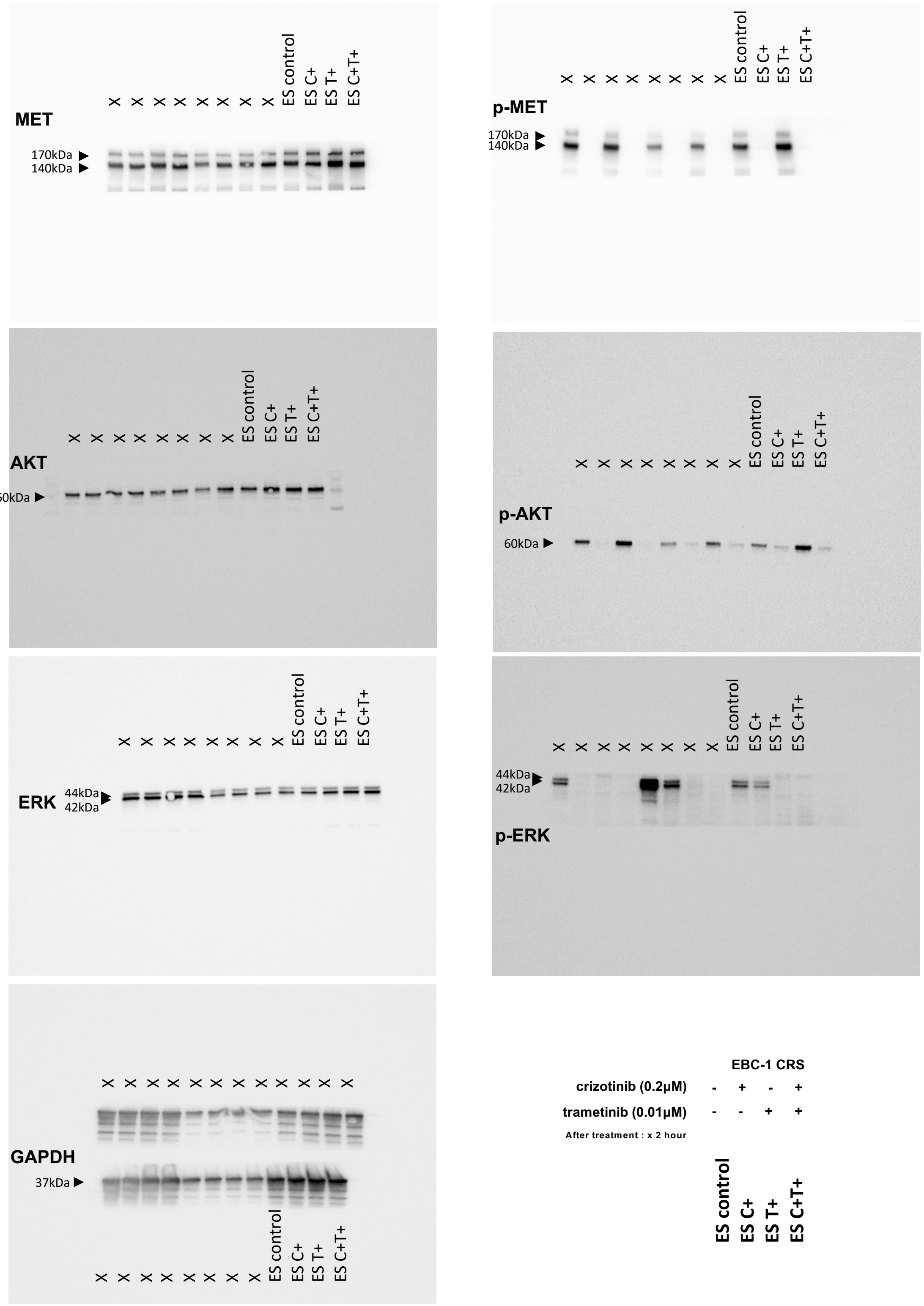
MET
170kDa
140kDa
ES control
ES C+
ES T+
ES C+T+
p-MET
170kDa
140kDa
AKT
60kDa
p-AKT
60kDa
ERK
44kDa
42kDa
p-ERK
44kDa
42kDa
GAPDH
37kDa
EBC-1 CRS
crizotinib (0.2μM) - + - +
trametinib (0.01μM) - - + +
After treatment : x 2 hour

Figure 3B

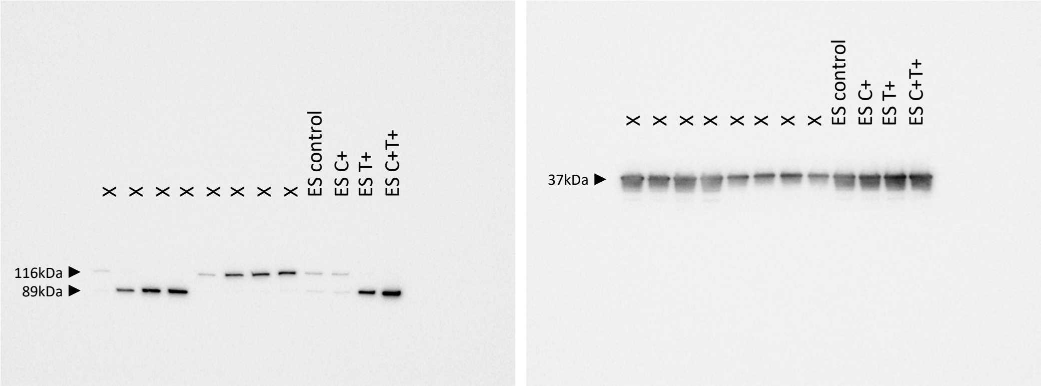

EBC-1 CRS

**crizotinib (0.2µM)**	-	+	-	+
**trametinib (0.01µM)**	-	-	+	+

After treatment : x 2 hour

ES control **ES C+** **ES T+** **ES C+T+**

	**EBC-1 Parental**		**EBC-1 CRH**		**EBC-1 CRS**	
**crizotinib (1µM)**						
**After treatment : x 2 hour**	-	+	-	+	-	+
	**EP-**	**EP+**	**EH-**	**EH+**	**ES-**	**ES+**

Figure 3I

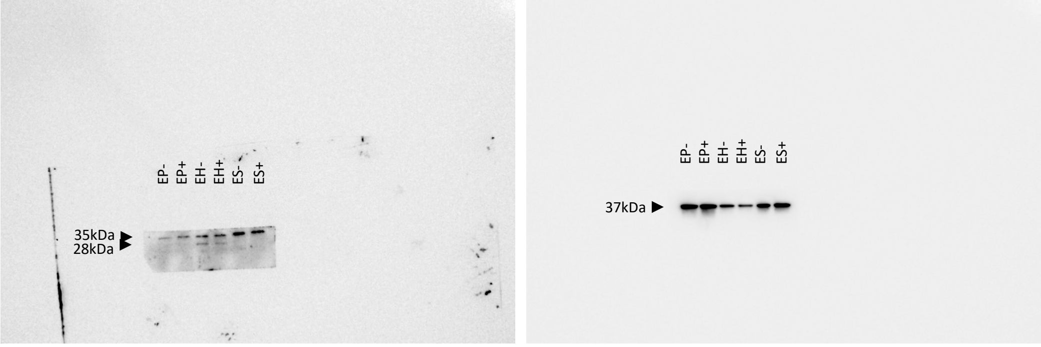

Figure 3E

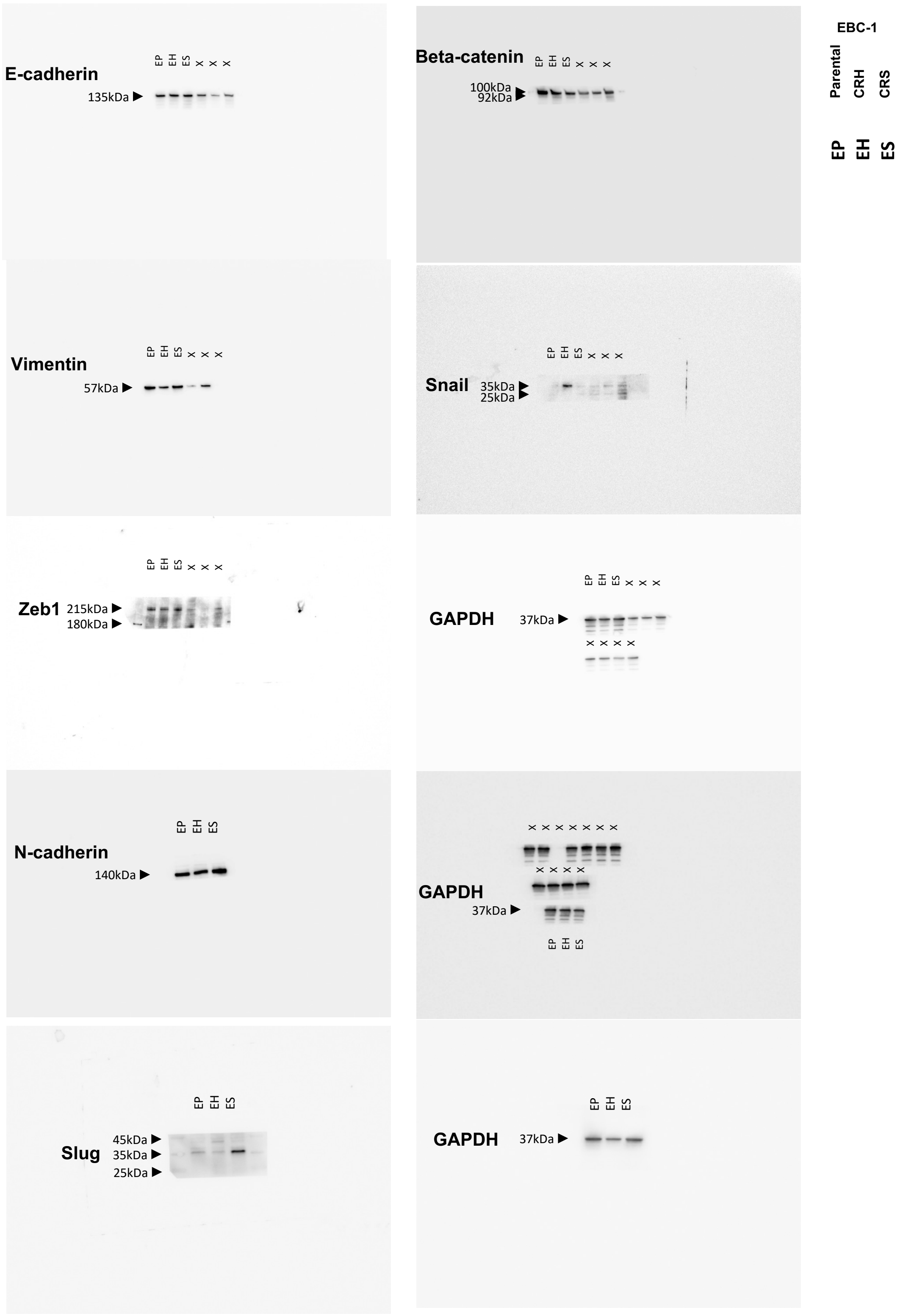
E-cadherin
EP EH ES × × ×
135kDa ▶
Beta-catenin
EP EH ES × × ×
100kDa ▶
92kDa ▶
EBC-1
Parental CRH CRS
EP EH ES
Vimentin
EP EH ES × × ×
57kDa ▶
Snail
EP EH ES × × ×
35kDa ▶
25kDa ▶
Zeb1
EP EH ES × × ×
215kDa ▶
180kDa ▶
GAPDH
EP EH ES × × ×
37kDa ▶
× × × ×
N-cadherin
EP EH ES
140kDa ▶
GAPDH
× × × × × × ×
× × × ×
37kDa ▶
EP EH ES
Slug
EP EH ES
45kDa ▶
35kDa ▶
25kDa ▶
GAPDH
EP EH ES
37kDa ▶

Figure 4A

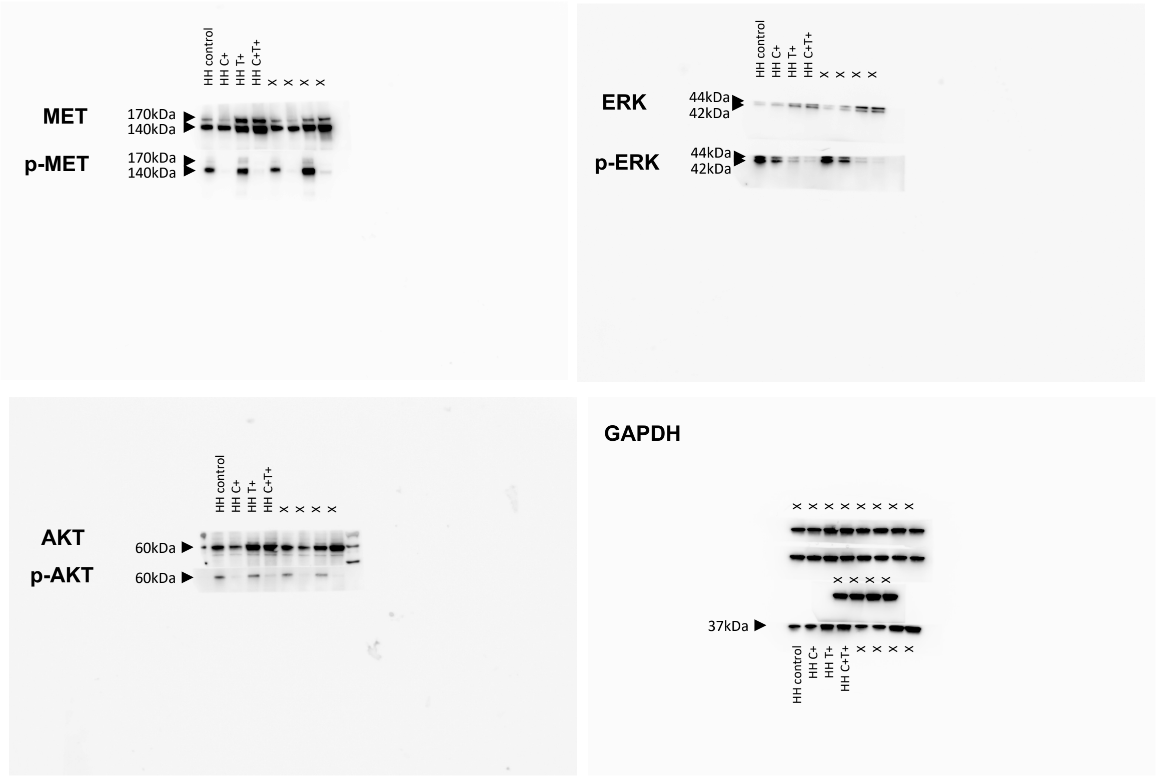

	H1993 CRH			
crizotinib (0.2μM)	-	+	-	+
trametinib (0.01μM)	-	-	+	+

After treatment : x 2 hour

HH control
HH C+
HH T+
HH C+T+

Figure 4B

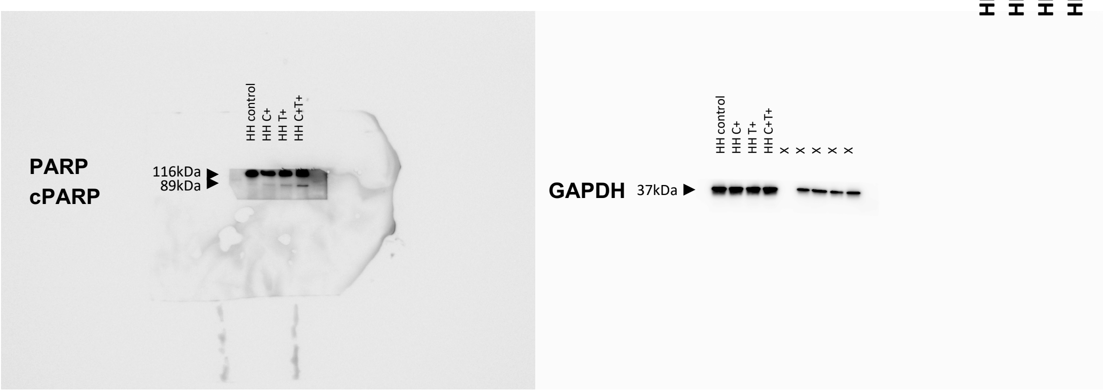

Figure 4G

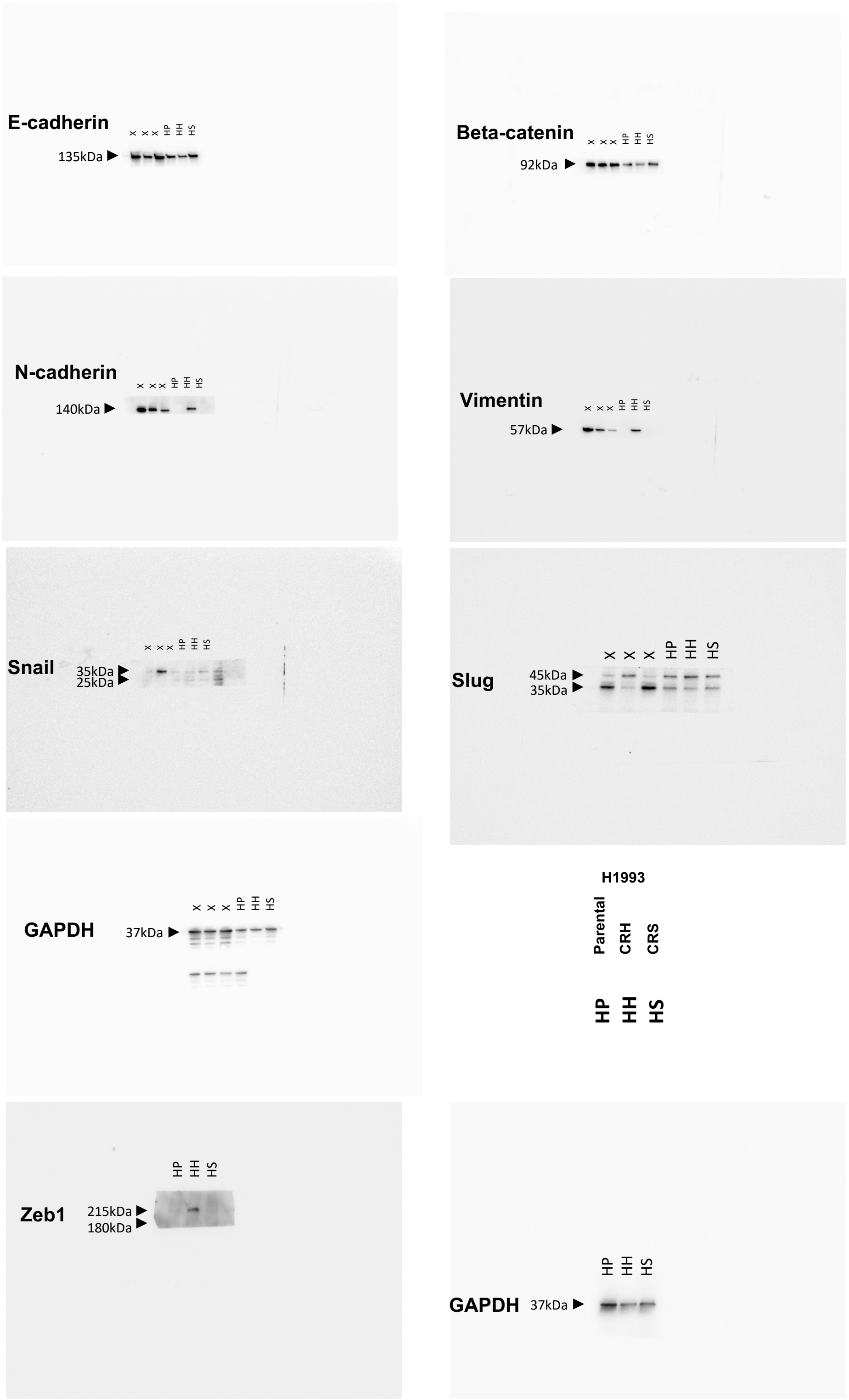

E-cadherin
X X X HP HH HS
135kDa
Beta-catenin
X X X HP HH HS
92kDa
N-cadherin
X X X HP HH HS
140kDa
Vimentin
X X X HP HH HS
57kDa
Snail
X X X HP HH HS
35kDa
25kDa
Slug
X X X HP HH HS
45kDa
35kDa
GAPDH
X X X HP HH HS
37kDa
H1993
Parental
CRH
CRS
HP
HH
HS
Zeb1
HP HH HS
215kDa
180kDa
GAPDH
HP HH HS
37kDa
