# Supplementary material for: PAI-1 mediates acquired resistance to MET-targeted therapy in non-small cell lung cancer
Source: PLoS One. 2024 May 17;19(5):e0300644. doi: 10.1371/journal.pone.0300644 (PMC11101109; doi:10.1371/journal.pone.0300644)
Supplement: Attachment — Submitted filename: Major concern.docx [file pone.0300644.s005.docx]

Major concern

1. Since all the cell viability assays are performed with six replicates, data for cell viability shown in Fig 1A need to be expressed with a standard deviation.
2. Signal transduction responses, shown in fig 1C, between two resistant-induced cell lines are different. The authors need to discuss the fundamental differences between these two induction processes and how they reflect on the clinical outcomes of patients.
3. The authors described that the SERPINE1 gene is six-fold higher in EBC-1 CRH cells. Please explain the disproportionate cell viability response in combination with MET-TKI and PAI-1 inhibitors (fig 1C).
4. There are no significant morphology differences between CRH and CRS in Fig 1B but substantial differences in the EMT-marker protein expression in Fig 3 and Fig 4. Please explain it.
5. It is not appropriate to conclude that EMT-induced antiapoptotic Bcl-2 expression in line 321-324 without any data support.
6. The authors need to conduct all the Western analyses of the EMT transition marker proteins with significant expression differences in gene analysis.
